# Histological validation of diagnoses of thyroid cancer among adults in the registries of Belarus and the Ukraine

**DOI:** 10.1038/sj.bjc.6601395

**Published:** 2003-11-25

**Authors:** B Franc, M Valenty, K Galakhin, E Kovalchuk, V Kulagenko, A Puchkou, Y Sidorov, M Tirmarche

**Affiliations:** 1 Service Anatomie et Cytologie Pathologiques, Hôpital Ambroise Paré, 92100 Boulogne, France; 2 Inserm U 494, 91, Boulevard de l'hôpital, 75013 Paris, France; 3 IRSN/DPHD/LEADS, BP 17, 92262 Fontenay aux Roses Cedex, France; 4 Institute of Oncology AMS of the Ukraine. Pathology Department, Lomonosova Str. 33/43, Kiev 03022, Ukraine; 5 Minsk City Anatomopathological Bureau, 8 Smeashko str, Bldg 5, 220089 Minsk, Belarus; 6 Minsk City Oncological Dispensary, 64F Skaryny Ave., 220600 Minsk, Belarus

**Keywords:** thyroid carcinoma, diagnostic, Belarus and Ukrainian registries

## Abstract

In order to evaluate the diagnostic reliability of the thyroid cancers listed in adult registries from the Ukraine and Belarus, a histological review was organised of 327 randomly selected thyroid carcinoma cases diagnosed between 1980 and 1999. A final diagnosis was reached at a 5-day consensus conference by six pathologists who met around a multiheaded microscope. The study concluded with a comparison between the final diagnosis and the initial diagnosis. The pathologists agreed with the initial diagnosis of malignancy in 286 cases (88%). A final diagnosis of papillary, follicular or medullary thyroid carcinoma was reached in 86, 4, and 6% of the cases respectively. In 2.8% of the cases reviewed, diagnostic discrepancies persisted. The percentage of agreement between the final diagnosis and the initial diagnosis was 93%, with a weighted *κ*-statistic of 0.61 (confidence interval 95% (CI_95%_): [0.45–0.77]). In all, 89% of the 286 confirmed cancer cases were in agreement for the type of cancer, with a *κ*-statistic of 0.56 (CI_95%_: [0.43–0.69]). The level of agreement differed according to cancer categories, with concordance rates of 94, 40 and 33% for papillary, follicular and medullary thyroid carcinomas respectively. The low prevalence of follicular thyroid carcinomas in the adult population studied calls for further exploration. The discrepancies and classification difficulties encountered were analysed.

The Chernobyl nuclear accident on 26 April 1986 led to the massive release of radionuclides into the environment. Although large areas of Europe were affected by Chernobyl-related ionising radiation, the accident had the greatest impact in Belarus, the Ukraine and parts of the Russian Federation. Epidemiological studies investigating the link between the Chernobyl accident and cancer incidence have mainly focused on malignant diseases in children, and specifically on thyroid cancer and leukaemia (Prisyazhiuk *et al*, 1991; Kazakov *et al*, 1992; Stsjazhko *et al*, 1995; Moysich *et al*, 2002). So far, no strong evidence has emerged to suggest that the risk of thyroid cancer has increased in the adult population as a result of the Chernobyl accident. Three studies published thyroid-cancer incidence rates after the Chernobyl accident in contaminated areas of the Ukraine and the Russian Federation (Prisyazhniuk *et al*, 1995; Ivanov *et al*, 1997; Ivanov *et al*, 1999) and they indicated that there had been no major change in adult thyroid-cancer incidence rates in either area.

In contrast to diagnoses reported in children or adolescents, no previously reported adult studies either in the Ukraine or Belarus have broken down pathological diagnoses of thyroid carcinoma into variants. The existence of cancer registries in those countries has made it possible to carry out epidemiological studies of thyroid cancer (Van Hoff *et al*, 1997; Tronko *et al*, 1999).

Epidemiological studies carried out in Western countries have usually relied on pathological reports, with no histological review of the cases (Gilliland *et al*, 1997). An absence of standardisation in the histological reports may lead to mistakes (Rigby *et al*, 1999; Branston *et al*, 2002; Leenhardt *et al*, 2003). Moreover, recent studies have mentioned diagnostic difficulties during the review of pathological findings in several cases of thyroid tumours in children and adolescents exposed to fallout from the Chernobyl accident, especially in encapsulated tumours with a follicular pattern (Williams, 2000).

A French-German initiative, in collaboration with the Ukraine, Belarus and Russia, has made it possible to carry out several studies related to the health consequences of the Chernobyl accident. Three studies dealt with the incidence of thyroid carcinoma in adults (15 years and over). In order to assess the reliability of the data provided by the initial reports, the pathology slides corresponding to the reports of thyroid carcinoma included in the registries of Belarus and the Ukraine between 1980 and 1999, were reviewed. A panel of six pathologists (from France, the Ukraine and Belarus) carried out the descriptive study. The results of the histological review of a series of 327 thyroid carcinomas in adults are presented in this descriptive study. The cases were randomly selected from the cancer registry files and reviewed by the panel. The initial diagnosis was compared to the panel's final consensus diagnosis. Diagnostic difficulties and discrepancies were analysed and compared to the data in the literature. The aim of the study was to determine the reliability of epidemiological studies based on pathological reports without any histological review of the malignant cases registered. In our study, we therefore analysed interobserver variation during the panel meeting, and the discrepancies between the initial and final diagnoses.

## MATERIAL AND METHODS

The thyroid carcinoma cases reviewed involved people at least 15 years of age and concerned the period 1980–1999. The cases were randomly selected from the files of the National Cancer Registry of Belarus (Vitebsk and Gomel oblasts or regions) and from the Database of the Ukrainian National Cancer Registry (Kiev, Zhitomir and Chernigov oblasts).

As a result of various difficulties in accessing material for the period prior to 1995, more cases were selected during the 1980–1994 period. In Belarus, 400 cases were selected in all (125 in each oblast for the period 1980–1994, 75 in each oblast for the period 1995–1999). In the Ukraine, 600 cases were selected (200 in Kiev and 100 in other oblasts for the first period, 100 in Kiev oblast and 50 in other oblasts for the second period). Out of these 1000 randomly selected cases, only 327 cases (199 from Belarus and 128 from the Ukraine) were reviewed. The slides of the remaining cases were not available, not retrieved, or of poor quality and these cases were therefore excluded from re-examination by the panel. All the cases provided by both Belarussian and Ukrainian pathologists were reviewed before the panel meeting. Even when the initial malignant diagnosis had been revised to that of a benign lesion, the report in the corresponding registry was not withdrawn.

The panel consisted of three Belarussian pathologists (V Kulagenko, A Puchkou and Y Sidorov), two Ukrainian pathologists (K Galakhin and E Kovalchuk) and one French pathologist (B Franc). All 327 cases were rendered anonymous before review. The consensus conference was held in Paris over a period of 5 days (Ambroise Paré Hospital, Boulogne, France), around a multiheaded microscope. The slides available (stained with haematoxylin and eosin) were re-examined and re-classified on the basis of the World Health Organization (WHO) classification as well as that of the Atlas of thyroid tumours of the Armed Forces from the Institute of Pathology (AFIP) (Hedinger *et al*, 1988; Rosai *et al*, 1992). Difficult cases of encapsulated follicular tumours were classified as atypical adenomas when a diagnosis of minimally invasive follicular thyroid carcinoma (MIFTC) was not obvious, or as a well-differentiated tumour of uncertain potential (WDT-UMP) in accordance with the Guest Editorial provided by Williams *et al* in 2000, when questionable papillary thyroid carcinoma-type (PTC) nuclear changes were encountered (Williams, 2000).

### Development of the study

The pathologists were first asked to assign the tumours to one of the major categories of thyroid tumours listed in Table 1

**Table 1:** Histological classification

**Malignant tumor**		**Malignancy excluded**		**Difficult diagnosis**
**Type**	**Subtype**	**Type**	**Subtype**	
Papillary carcinoma	Follicular variant	Adenoma	Oxyphilic type	Atypical adenoma
	Encapsulated variant		Other	Well-differentiated tumour of uncertain malignant potential (WDT-UMP)
	Invasive variant	Goitre		
	Tall cell variant			
	Columnar cell			
	Microcarcinoma			
	Oxyphilic cell type			
	Clear cell			
	Warthin like			
				
Follicular carcinoma	Minimally invasive (encapsulated)			
	Widely invasive			
	Oxyphilic cell type			
	Clear cell type			
				
Poorly differentiated carcinoma				
Anaplastic carcinoma				
Medullary carcinoma				
Malignant lymphoma				
Metastases				
Other carcinoma				

. They were also asked to subtype the different carcinomas diagnosed in accordance with the subcategories listed in Table 1.

In the second phase, the cases in which discrepancies occurred during the panel conference were re-examined at the end of the panel in order to reach a consensus diagnosis.

The last phase was to examine the agreement between the initial diagnosis, made in the Ukraine or Belarus, and that reached by the panel. Reliability was evaluated at two levels: the accuracy of the malignancy or malignancy-excluded diagnosis, and the accuracy of the malignancy categories. The level of agreement between the initial diagnosis and the final consensus was evaluated using the *κ*-statistic (Agresti, 1990). The level of agreement between the initial diagnosis and the final consensus was evaluated using the *κ*-statistic for qualitative data, which is a test commonly used to evaluate the concordance between qualitative data. We used the following threshold values for the level of agreement: <0.00, poor; 0.00–0.20, slight; 0.21–0.40, fair; 0.41–0.60, moderate; 0.61–0.80, substantial; 0.81–1.00, almost complete. For the ordinal values, we used the weighted *κ*-statistic as recommended in the literature (Cohen, 1960; Landis and Koch, 1977).

## RESULTS

### Panel consensus conference

The population whose slides were examined consisted of 246 female and 81 male subjects. The minimum age was 15 years and the maximum was 82 years, with a mean age of 45 years. The final panel consensus excluded one case because insufficient material was available. A total of 326 cases was therefore analysed.

Malignancy was excluded in 29 cases (9%). The distribution of the various cancer categories is listed in Table 2

**Table 2:** Distribution of cancer (297) according to region (panel diagnosis)

**Region**	**Papillary carcinoma**	**Follicular carcinoma**	**Poorly differentiated carcinoma**	**Medullary carcinoma**	**Malignant lymphoma**	**Paraganglioma**	**Metastasis**	**Unclassified tumour**
Gomel (Belarus) (112)	99 (88%)	4 (4%)	0 #	7 (6%)	1 (1%)	1 (1%)	0 #	0 #
Vitebsk (Belarus) (71)	63 (89%)^a^	3 (4%)	1 (1%)	2 (3%)	0 #	0 #	2 (2%)	0 #
Chernigov (The Ukraine) (26)	19 (73%)	4 (15%)	0 #	1 (4%)	1 (4%)	0 #	1 (4%)	0 #
Kiev (The Ukraine) (64)	55 (86%)^a^	0 #	1 (2%)	7 (11%)	0 #	0 #	0 #	1 (2%)
Zhitomir (The Ukraine) (24)	20 (83%)	0 #	2 (8%)	1 (4%)	0 #	0 #	1 (4%)	0 #
								
Total	256 (86%)	11 (4%)	4 (1%)	18 (6%)	2	1 #	4 (1%)	1 #

a Anaplastic carcinoma.

. The distribution of the thyroid carcinoma types was similar in the different oblasts.

The six pathologists reached a consensus on 310 cases (95%). At the end of the panel conference, discrepancies persisted in nine out of 326 cases (2.8%).

### Carcinoma subcategories and associated lesions

Of the 256 papillary carcinomas, 20 cases were encapsulated, 226 invasive, and in eight cases, there was no normal thyroid tissue counterpart. Among the 20 encapsulated papillary carcinomas, 45% were follicular variants. The several variants reported by the panel for papillary carcinomas are listed in Table 3

**Table 3:** Panel papillary carcinoma, variants (panel diagnosis)

**Oblast**	**Invasive**	**Follicular variant**	**Tall cells or columnar cells**	**Microcarcinoma**	**Oxyphilic cell type**	**Poorly differentiated (trabecular or solid)**	**Other papillary**
Gomel (Belarus) (99)	53 (47%)	27 (24%)	4 (4%)	5 (4%)	2 (2%))	4 (4%)	4 (4%)
Vitebsk (Belarus) (63)	27 (38%)	19 (27%)^a^	2 (3%)	6 (8%)	1 (1%)	3 (4%)	5 (7%)
Chernigov (The Ukraine) (19)	13 (50%)	4 (15%)	0 #	0 #	0 #	1 (4%)	1 (4%)
Kiev (The Ukraine) (55)	44 (69%)	4 (6%)	0 #	2 (3%)	0 #	2 (3%)	3^*^ (5%)
Zhitomir (The Ukraine) (20)	17 (71%)	1 (4%)	0 #	0 #	0 #	1 (4%)	1 (4%)
							
Total	154 (60%)	55 (21%)	6 (2%)	13 (5%)	3 (1%)	11 (4%)	14 (5.5%)

a Anaplastic carcinoma.

. The distribution of cancers according to the age at diagnosis is listed in Table 4

**Table 4:** Distribution of cancers according to age at diagnosis (panel diagnosis)

	**Age (years)**				
**Carcinoma**	**15–24 (15–18)**	**25–39**	**40–54**	**55–69**	**70 and above**
Total	33 (19)	68 #	101 #	84 #	11 #
					
Papillary carcinoma	31 (94%)	54 (79%)	88 (87%)	74 (88%)	9 (82%)
Follicular carcinoma	1 #	5 #	2 #	2 #	1 #
Poorly differentiated	0 #	0 #	2 #	2 #	0 #
Medullary carcinoma	0 #	6 #	7 #	5 #	0 #
Malignant lymphoma	1 #	1 #	0 #	0 #	0 #
Paraganglioma	0 #	1 #	0 #	0 #	0 #
Metastasis	0 #	1 #	2 #	0 #	1 #
Unclassified tumour	0 #	0 #	0 #	1 #	0 #

.

Concomitant lesions comprised 26 goitres and 130 cases of lymphocytic thyroiditis. Papillary carcinomas were combined with thyroiditis in 124 out of 256 cases (48%), follicular carcinomas were combined with thyroiditis in two out of 11 cases (18%), and medullary carcinomas combined with thyroiditis in four out of 18 cases (22%).

### Malignancy excluded

Among the 29 cases in which malignancy was excluded, 14 were difficult to classify as either benign or malignant. Five of these cases were classified as atypical adenomas and nine as WDT-UMP. The other nine cases corresponded to adenomas (two out of nine oxyphilic). The last six cases were hyperplastic nodules (five out of six in the context of severe lymphocytic thyroiditis).

### Discrepancies

In seven cases, a consensus was finally reached, and concerned:

- three malignant trabecular carcinomas, which were difficult to assign to either the follicular or papillary carcinoma group,
- one anaplastic carcinoma, in which the papillary counterpart was partly underidentified,
- three cases, which were finally diagnosed as adenomas, but which was difficult to assign to the adenoma or to WDT-UMP group.

In nine other cases (2.8%), the divergence persisted.

In five encapsulated cases, the diagnostic uncertainty concerned whether they were benign or malignant. The final diagnoses proposed were one atypical adenoma, two WDT-UMP, one trabecular encapsulated PTC, and one PTC follicular variant.

The remaining four cases concerned malignancy categories. A diagnosis of medullary thyroid carcinoma (MTC) was debated for three cases and this diagnosis was finally proposed in two cases, and one of oxyphilic papillary carcinoma in the third. This last case was located in a lymph node, and was discussed as a possible PTC *vs* a metastasis from another site; before being diagnosed as a PTC.

### Agreement between the initial diagnosis and the final consensus

Out of 326 cases, agreement was reached in 286 cases with regard to the diagnosis of malignancy (88%); one undifferentiated thyroid carcinoma diagnosis was considered to be impossible to classify to any category, one case had no initial diagnosis (Table 5

**Table 5:** Agreement between the initial diagnosis and the panel diagnosis

	**Panel diagnosis**			
**Initial diagnosis**	**Malignancy**	**Nonmalignancy**	**Doubtful diagnosis**	**Total**
Malignancy	286	5	9	300
Nonmalignancy	7	0	0	7
Doubtful Diagnosis	2	0	15	17
				
Total	295	5	24	324

). The percentage of agreement between the initial diagnosis and the final consensus was 93%, and the value of the weighted *κ*-statistic was 0.61 (confidence interval 95% (CI_95%_): [0.45–0.77]), which was considered substantial.

Among the 286 cancer cases, there was agreement in 254 with regard to the type of cancer (89%), and divergence about the other 32. In 16 of these 32 cases the diagnosis was of follicular carcinoma as in the previous diagnosis (4.9%) (Table 6

**Table 6:** Agreement between the initial diagnosis and the panel diagnosis for the confirmed cancers

	**Panel diagnosis**				
**Initial diagnosis**	**Papillary carcinoma**	**Follicular carcinoma**	**Medullary carcinoma**	**Poorly differentiated and others cancers**	**Total**
Papillary carcinoma	232	4	4	2	242
Follicular carcinoma	15	6	0	1	22
Medullary carcinoma	0	0	12	1	13
Poorly differentiated and other cancers	3	0	2	4	9
					
Total	250	10	18	8	286

). The *κ*-coefficient was 0.56 (CI_95%_: [0.43–0.69]), which was considered moderate.

## DISCUSSION

Most of the registry data rely on pathology reports with no review. After the Chernobyl accident, most of the published data concerning childhood thyroid pathology relied either on a direct examination of the pathological slides or were taken directly from the registry data. After reviewing several hundred cases of thyroid tumours, the Chernobyl pathologist group published a guest editorial to draw attention to some of the problems that have arisen in applying the WHO classification, and to the solutions that were adopted (Williams, 2000).

Reliability studies of the diagnoses of malignant thyroid tumour in different registries have already been performed for both child or adult carcinomas (Holm *et al*, 1980; Harach and Williams, 1995). In the study in Sweden by Holm *et al*, pathologists agreed with the registry diagnosis of malignant tumour in 85–94% of the cases (depending on the registry region). In the childhood registries from Wales and England, the reliability was 92%. None of these series used *κ*-statistics. In our study, the percentage of agreement between the initial diagnosis and the final consensus was 93%; the weighted *κ*-statistic value was 0.61 (CI_95%_: [0.45–0.77]); moreover, among the 286 cancer cases confirmed during the panel meeting, the percentage of agreement was 89% and the *κ*-coefficient was 0.56 (CI_95%_: [0.43–0.69]).

The various difficulties encountered in thyroid tumour diagnosis needed to be reviewed.

The reproducibility of histological classification in thyroid carcinoma has previously been addressed in a few studies (Saxen *et al*, 1978; Holm *et al*, 1980; Fassina *et al*, 1993; Franc *et al*, 1999; Hirokawa *et al*, 2002). In most of these studies, the review of the cases was performed separately by each observer. This contrasted with our study, where each case was reviewed simultaneously by all pathologists around a multiheaded microscope.

In our series, majority agreement was reached in a high percentage of cases (95%), probably as a result of the predominance of PTC with its usual characteristics. However, as in Fassina's study on cancer subclassification (Fassina *et al*, 1993), several debates occurred during our consensus conference when we were reviewing PTC subtypes and encapsulated thyroid follicular tumours. The level of agreement differed depending on the cancer category from 94% for PTC and 40% for follicular thyroid carcinoma (FTC).

It is important to note the increased diagnosis of papillary carcinoma follicular variant in the last decade, in adult, childhood and adolescent thyroid cancer (Harach and Williams, 1995; Pacini *et al*, 1997; Tronko *et al*, 1999; Verkooijen *et al*, 2003). Even though there have been improvements in diagnostic practice that have affected the incidence of this type of cancer, this may be explained by changes that have occurred in histological definitions. The present rules were progressively adopted at the end of the 1980s and the beginning of the 1990s. As a result, a large proportion of cancers, that would previously have been classified as follicular carcinomas or mixed papillary follicular carcinomas are now classified as PTC because of nuclear PTC features (Colonna *et al*, 2002; Verkooijen *et al*, 2003). Certain cases that were formerly classified as benign (and therefore not included in the cancer incidence figures) have now been classified as papillary carcinoma (Leenhardt *et al*, 2003).

In our study, we were surprised by the low frequency of FTC (both in the initial diagnosis and in the final consensus). This FTC frequency is in the same range as in the Ukrainian registry series or children (5.3%) (Tronko *et al*, 1999). These low incidences of FTC among children and adults contrast with those in Western countries (11–15.2%) (Harach and Williams, 1995; Pacini *et al*, 1997). The frequency of FTC in our study contrasts with that in the Ukrainian registry 1998–2000 (16% for FTC) (Fedorenko *et al*, 2002), and with that of Hundahl *et al* (1998). Some of the FTC (68%, Table 6) cases were assigned by the panel to the PTC group or to the intermediate tumour group (atypical and WDT-UMP). Some of these diagnostic difficulties already encountered in Western countries (Franc *et al*, 1999; Williams, 2000; Hirokawa *et al*, 2002) can interfere with the determination of the true prevalence of FTC.

Are these lower frequencies of FTC, or rather the higher frequencies of PTC, in the Ukraine and Belarus an outcome of the Chernobyl accident or, as demonstrated recently elsewhere, attributable to a change in histological criteria (Leenhardt *et al*, 2003; Verkooijen *et al*, 2003)?

These conflicting conclusions may be due to the insufficient number of cases studied. A larger sample of thyroid carcinomas must be analysed to find out whether the FTC group is under-represented in our series and, if so, to what extent.

There have been serious efforts in contaminated areas to validate the registry data by reviewing the pathology samples. This is not the case in large international epidemiological studies, which have relied on pathology reports with no review of tissue specimens (Hundahl *et al*, 1998; Holzer *et al*., 2000; Hundahl *et al*, 2000).

Our study shows that these two registries are as reliable as those in Western countries. It highlights the validity of registries when considering invasive carcinomas, whether they are differentiated or not, but underlines the low reproducibility of diagnoses of encapsulated follicular tumour. Reproducibility studies must be encouraged in order to propose better diagnostic guidelines for diagnosis, and to confirm the validity of data registries when they rely on histological reports. The size of the randomly selected studied sample must be appropriately determined.

The low prevalence of FTC in the adult population studied walls for further exploration.
